# Acute and Chronic Oral Toxicity of a Partially Purified Plaunotol Extract from *Croton stellatopilosus* Ohba

**DOI:** 10.1155/2013/303162

**Published:** 2013-09-30

**Authors:** Chatchai Chaotham, Songpol Chivapat, Anan Chaikitwattana, Wanchai De-Eknamkul

**Affiliations:** ^1^Pharmaceutical Technology (International) Program, Faculty of Pharmaceutical Sciences, Chulalongkorn University, Phayathai Road, Bangkok 10330, Thailand; ^2^Medical Plant Research Institute, Department of Medical Sciences, Ministry of Public Health, Tivanond Road, Nonthaburi 11000, Thailand; ^3^Department of Biotech Business, Tipco Biotech Co. Ltd., Phetkasem Road, Prachuap Khiri Khan 77000, Thailand; ^4^Department of Pharmacognosy and Pharmaceutical Botany, Faculty of Pharmaceutical Sciences, Chulalongkorn University, Phayathai Road, Bangkok 10300, Thailand

## Abstract

Plaunotol, an acyclic diterpenoid with highly effective antigastric ulcer properties, has been commercially isolated from leaves of *Croton stellatopilosus* Ohba. This Thai medicinal plant was traditionally used in the form of crude extracts, suggesting that it is possible to administer these plaunotol-containing extracts without toxicity. To confirm its safety, the oral toxicity of a partially purified plaunotol extract (PPE) was evaluated *in vivo*. The PPE was simply prepared by 95% ethanol reflux extraction followed by hexane partition. The obtained extract was analyzed and found to contain 43% w/w of plaunotol and another compound, likely a fatty acid-plaunotol conjugate that is considered a major impurity. Oral administration of PPE to ICR mice and Wistar rats was conducted to evaluate acute and chronic toxicity of the plaunotol extract, respectively. The acute toxicity study demonstrated that PPE was practically nontoxic based on its high median lethal dose value (LD_50_ = 10.25 g/kg). The chronic toxicity studies also showed the absence of mortality and clinical symptoms in all rats treated with 11–1,100 mg/kg/day of PPE during a 6-month period. Histopathological and hematological analyses revealed that altered liver and kidney function and increased blood platelet number, but only at the high doses (550–1,100 mg/kg/day). These results suggest that PPE is potentially safe for further development as a therapeutic agent in humans.

## 1. Introduction

*Croton stellatopilosus* Ohba, a Thai medicinal plant (Plau-noi), has been acknowledged for its traditional remedy in the treatment of helminthes and topical infection [1]. A major constituent isolated from leaves of Plau-Noi is plaunotol [2]. This natural acyclic diterpene has been used as an antigastric ulcer medication based on its pharmacotherapeutic effects in inducing prostaglandin E_2_ (PGE_2_) and eradicating *Helicobacter pylori* bacteria [3–6]. However, in addition to its application as a single compound that requires a complicated process of extraction and purification [7], plaunotol used in form of a partially purified plaunotol extract (PPE) is also of interest. This is due to the wide distribution of Plau-Noi plant in Thailand [8] and the reliability of pharmacological benefits of natural terpenoid provides in human healthcare [9, 10]. Furthermore, the use of PPE can minimize the cost of production and increase the access to an effective drug at a low price.

At a dose of 240 mg/day or 4.8 mg/kg/day, plaunotol has been recommended to achieve antigastric ulcer effects in humans [11]. Because equivalent pharmacological activities could potentially come from higher amounts of PPE than those of pure plaunotol, it is necessary to assess toxicity profiles of such a high doses of PPE. Therefore, this study aims to evaluate oral toxicity of PPE in order to generate a safety data in animal models for extrapolation to human toxicity profiles.

## 2. Materials and Methods

### 2.1. Preparation of PPE

PPE was prepared from dried leaf powder of *Croton stellatopilosus *Ohba by modifying a previously described method [7]. The leaf powder was refluxed with 95% ethanol (1 : 1 ratio by weight) at 70°C for 1 h. After filtration, the extract was concentrated by using a rotary evaporator, followed by partitioning with hexane (ratio of 1 : 1) to remove highly nonpolar constituents. With plaunotol remaining in ethanol layer, the ethanol was evaporated in a vacuum to obtain PPE, which was then collected and stored at 4°C. For use in this toxicity study, PPE was suspended in a 0.5% tragacanth solution and adjusted to various concentrations as indicated. 

### 2.2. Plaunotol Content Analysis

Quantitative analysis of plaunotol in PPE was conducted using the method of thin-layer chromatography (TLC) reported previously [12, 13]. Briefly, a silica gel 60 F_254_ TLC plate (Merck, Darmstadt, Germany) was spotted with PPE and standard plaunotol (obtained from Thai Sankyo Co., Ltd., which is currently under Tipco Biotech Co. Ltd., Prachuab Khiri Khan, Thailand). The plate was then developed using a solvent system of chloroform:n-propanol (96 : 4). The developed TLC plate was scanned by a densitometer at 220 nm using a Camag TLC Scanner 3 with Wincats software V 1.3.4 (Camag, Muttenz, Switzerland). Structural analysis of each constituent was carried out using gas chromatography-mass spectrometry (GC-MS: Agilent Model 6890N-5973N, Agilent Technologies, CA, USA). A DB-5 capillary column (30 × 0.25 mm) with helium as a carrier gas at a flow rate of 13.8 mL/min was heated from 150°C up to 300°C with an increasing temperature rate of 15°C/min. Temperatures of both detector and the injector were set at 250°C [12]. Compound identification was performed by comparing the resulting mass spectra with those available in the Wiley Registry of Mass Spectral Data 7th edition (McLafferty, 2000), Agilent Part no. G1035B.

### 2.3. Animals and Housing

 All ICR (Imprinting Control Region) mice and Wistar rats were obtained from the National Laboratory Animal Center (Mahidol University, Nakorn Pathom, Thailand). They were housed in the conventional hygienic animal rooms of the Laboratory Animal Center, Department of Medical Sciences, Nonthaburi, Thailand. Room conditions were maintained at 25°C, 60% humidity and 12 hour-light-dark cycle. The animals were given a commercial pellet diet 082 CP feed (Perfect Companion Group, Thailand) and clean water ad libitum. Prior to the experiment, the animals were allowed two weeks to acclimate to the environment.

### 2.4. Acute Toxicity Study

Five male and five female ICR mice were treated with either water for the control group or PPE at 2.5, 5, 10, and 20 g/kg for each treatment group. All animals were food restricted for 2 h prior to oral administration of single doses of the substances. Observations of clinical presentation and mortality were conducted at 15 min, 30 min, 3 h, 6 h, and 24 h following treatment and daily for 14 days thereafter. Finally, the mean lethal dose value (LD_50_) was calculated. This study was authorized by the Institutional Animal Care and Use Committee, Department of Medical Sciences as Permission no. 52-002.4. 

### 2.5. Chronic Toxicity Study

The protocol used for the chronic toxicity study was approved by the Institution of Animal Care and Use Committee, Department of Medical Sciences as Permission no. 53-014. Wistar rats were randomized into six groups, each with 15 male and 15 female rats. Four experimental groups were administered the PPE suspension orally at doses of 11, 110, 550, and 1100 mg/kg/day for six months. Two control groups received distilled water and 0.5% tragacanth solution orally at the volume of 10 mL/kg. During the experimental period, animals were observed daily for general appearance and signs of toxicity. Body weight and food consumption were measured weekly. At the end of the study, animals were fasted overnight and were then killed using diethyl ether inhalation. Blood samples were collected from the posterior vena cava for hematological and biochemical value measurements. 

Hematological analysis was performed using the hematological analyzer Cell Dyn 3500 (Abbot Laboratories Ltd., IL, USA). The parameters examined included red blood cells (RBC), hematocrit (Hct), hemoglobin, mean cell volume (MCV), mean cell hemoglobin (MCH), mean cell hemoglobin concentration (MCHC), white blood cells (WBC), lymphocytes, neutrophils, eosinophils, basophils, monocytes, and platelets. Biochemical values were measured using Cobas integra 400 (Hoffmann-La Roche Ltd., Basel, Switzerland), which assessed levels of alkaline phosphatase (ALP), alanine aminotransaminase (ALT), aspartate aminotransaminase (AST), total protein, albumin, bilirubin, blood urea nitrogen (BUN), creatinine, glucose, uric acid, triglyceride, cholesterol and sodium, potassium, and chloride ions. 

A complete necropsy was performed to assess gross lesions of visceral organs. Brain, lung, heart, liver, kidney, stomach, spleen, testis, uterus, urinary bladder, and adrenal glands were isolated and weighed using Mettler (Mettler Toledo PB 153 balance, Mettler Toledo Intl. Inc., Zurich, Switzerland). Relative organ weight was calculated. The above mentioned organs, including trachea, were fixed in 10% phosphate-buffered formalin and subjected to conventional histological processing and stained with hematoxylin for further histopathological examination [14].

### 2.6. Statistical Analysis

A one-way ANOVA was used to evaluate significant differences between groups via the Bonferroni test. Fisher's exact was applied to compare histopathological differences between groups. *P* ≤ 0.05 was considered statistically significant.

## 3. Results

### 3.1. Plaunotol and a Plaunotol-Like Compound Are Major Constituents of PPE

**Figure 1:**
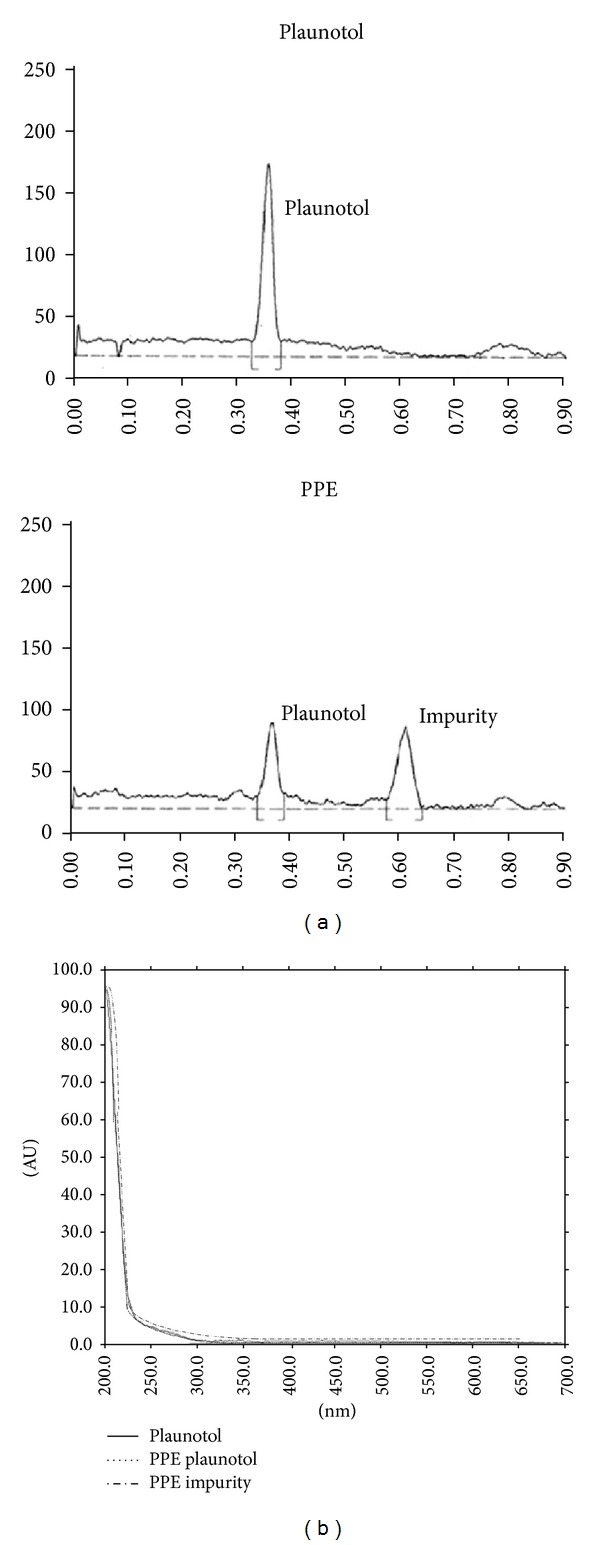
Densitometric TLC for separation of PPE constituents in PPE and quantitative analysis of plaunotol. (a) TLC chromatogram of PPE showing two major peaks of plaunotol and a main impurity. (b) Comparison of the UV absorption spectra obtained from the wavelength scanning at the spots of standard plaunotol, PPE plaunotol, and the PPE impurity shown in (a).

**Figure 2:**
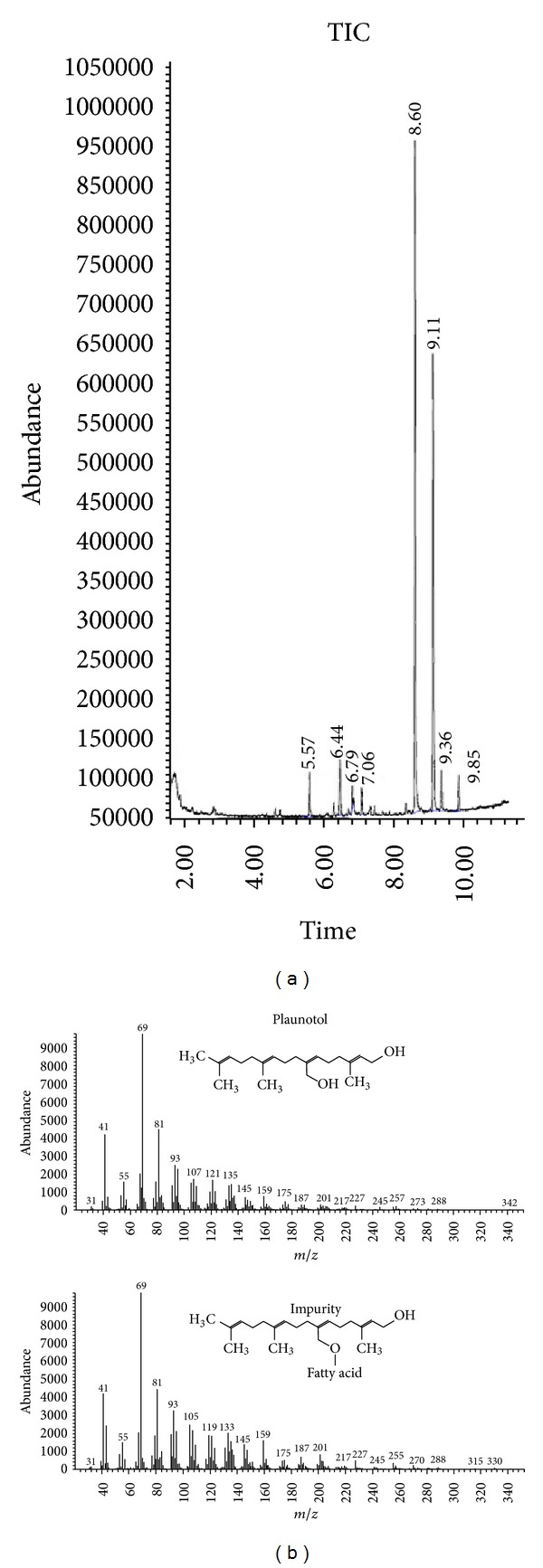
GC-MS chromatogram of PPE (a) and the mass spectra (b) of the two major peaks of plaunotol and the impurity identified based on the total ion chromatograms (TIC) of plaunotol and the major impurity in PPE.

By modifying the conventional method of plaunotol extraction and limited purification, PPE was obtained in 2% yield (w/w) as a brownish-yellow translucent oil. The extract appeared to contain plaunotol and an unknown compound as the two major constituents and a few minor impurities, as shown by the chemical profiles in the TLC (Figure 1(a)) and GC (Figure 2(a)) chromatograms. The UV absorption spectrum (Figure 1(b)) and mass spectrum (Figure 2(b)) of the unknown compound were similar to those of plaunotol, suggesting that the major impurity in PPE was a plaunotol-like compound. Because fatty acid conjugates of plaunotol are known to be present in the leaves of *C. stellatopilosus* [15], it is likely that the major impurity was one of the fatty acid-plaunotol conjugates that were extracted with plaunotol. Quantitative analysis indicated that plaunotol was present at approximately 43.0% w/w in the PPE preparation. 

### 3.2. Acute Oral Toxicity and LD_50_

All of the observed PPE-induced acute toxicity symptoms are summarized in Table 1. The results were based on oral doses of PPE at 2.5, 5, 10, and 20 g/kg, calculated to be 200, 400, 800, and 1,600 times the recommended dose of plaunotol for human therapeutic use, respectively [11]. Acute diarrhea was detected early, 6 h after extract administration, at all doses and remained until 72–96 h. With the high doses of 10 and 20 g/kg, other clinical symptoms were observed, including dyspnea (shortness of breath), dullness, and stomach cramp. 

Mortality was not observed in mice treated with water (control) or PPE at 2.5 and 5 g/kg; however, it was observed in four (one male and three females) and 10 (five males and five females) mice between 24 and 72 h at the 10 and 20 g/kg doses, respectively. Probit analysis revealed that the LD_50_ of PPE was 10.25 g/kg with 95% confidence limit between 7.37 and 14.63 g/kg. 

### 3.3. Chronic Oral Toxicity

#### 3.3.1. Effect of PPE on Body Weight, Food Consumption, and Health Status

**Figure 3:**
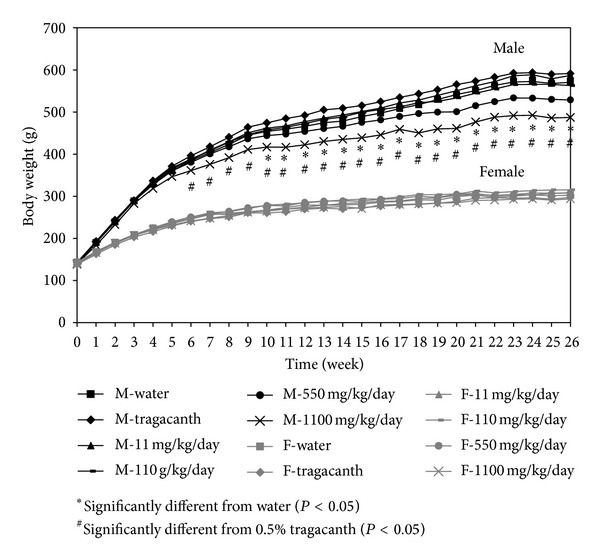
Growth curves of the male and female rats receiving PPE for 6 months.

**Figure 4:**
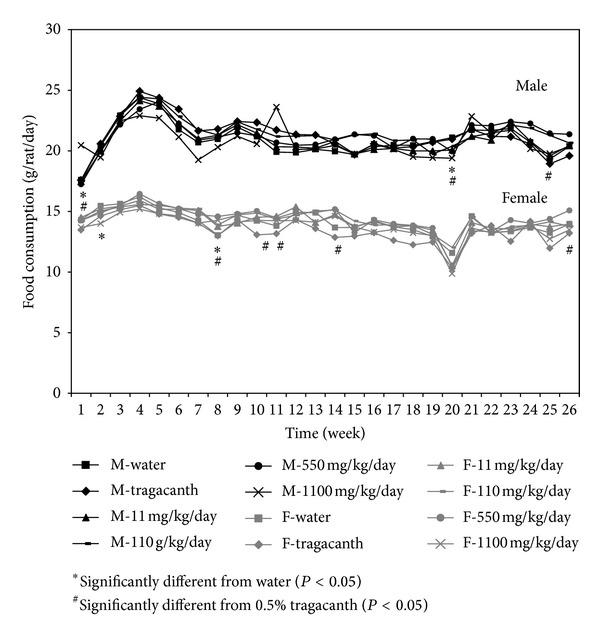
Food consumptions for male and female rats receiving PPE for 6 months.

In this chronic toxicity study, rats were fed equal volumes of PPE to minimize the effect of administration differences. PPE was diluted in 0.5% w/w of tragacanth to achieve the desirable concentrations used for gavage feeding the rats. The selected concentrations of PPE were 11, 110, 550, and 1,100 mg/kg/day, which are equivalent to 1, 10, 50, and 100 times the therapeutic dose of plaunotol, respectively. Mortality and significant clinical symptoms were not observed in any of the rats fed with PPE, water, and 0.5% w/w tragacanth (Figure 3). It is important to note that, for male rats given the high dose of PPE at 1,100 mg/kg/day, a trend in altered food consumption was not apparent even though there was a significant decrease in body weight (Figure 4). Similarly, the body weight and food consumption among female rats showed no significant difference.

#### 3.3.2. Effect of PPE on Relative Organ Weight Values

 The values of relative organ weight, expressed as g/1000 g body weight, are summarized in Tables 2 and 3. For male rats, relative organ weight values of the heart, liver, stomach, spleen, and kidney were significantly increased at doses of 110, 550, and 1,100 mg/kg/day, compared with the water and tragacanth control groups (Table 2). Furthermore, relative weights of the brain, lung, bladder, adrenal gland, and testis were also higher in the male rats given 550 and 1,100 mg/kg/day of PPE.  Table 3 summarizes the values of relative organ weight for female rats. It can be seen that PPE treatment at high doses (550 and 1,100 mg/kg/day) caused an increase in the relative weight of the liver and kidney compared to both control groups. An additional organ that appeared to be affected in the female group was the stomach, which showed significantly higher relative weights in the 550 and 1,100 mg/kg/day PPE treatment groups.

#### 3.3.3. Effect of PPE on Altered Hematological Values

 After feeding PPE at 11 and 110 mg/kg/day for 6 months to both male and female rats, no significant alterations in any of the hematological values were observed (Tables 4 and 5). At the high doses of 550 and 1,100 mg/kg/day, male rats had significantly higher platelet numbers but a lower percentage of eosinophils compared to both controls. The hematocrit, RBC, and hemoglobin values were significantly higher only in the 1,100 mg/kg/day treatment group, although their MCHC was significantly lower. 

The reduced value of %eosinophil was observed in female rats administered 550 and 1,100 mg/kg/day of PPE. These two groups also appeared to have significantly higher values of platelet numbers and percentage of basophil compared to both controls. Moreover, the female rats treated with 1,100 mg/kg/day had significantly lower MCV, MCH, and MCHC compared to the controls, while the RBC and WBC values were significantly higher. 

#### 3.3.4. Effect of PPE on Clinical Chemistry Values

 An increase in blood glucose levels was observed in male rats treated with PPE at 11 to 550 mg/kg/day compared to the water control group. Surprisingly, only the male rats fed 1,100 mg/kg/day showed lower blood glucose levels (Table 6), whereas the female rats had higher blood glucose levels following treatment with 110 to 1,100 mg/kg/day of PPE, compared to the tragacanth control group (Table 7).

Increased ALP values were observed in male rats that received 110 to 1,100 mg/kg/day and in female rats that received the highest dose of 1,100 mg/kg/day. Decreased AST levels were observed only at the 550 mg/kg/day dose in both genders of rat. Total protein level was significantly increased in the male rats receiving 11 and 550 mg/kg/day, as well as in female rats receiving 550 and 1,100 mg/kg/day. Increased albumin and BUN levels were observed in both male and female rats given 550 and 1,100 mg/kg/day of PPE compared to both control groups. 

The cholesterol values were significantly higher in both male and female rats that received PPE at 550 and 1,100 mg/kg/day. In contrast, male rats treated with 1,100 mg/kg/day of the extracts had lower triglyceride levels compared to both water and tragacanth control groups. Lower chloride levels were observed in male rats given 110 and 550 mg/kg/day and female rats given 550 and 1,110 mg/kg/day. High levels of uric acid were measured in male rats, but only at the doses of 11 and 550 mg/kg/day. Female rats receiving PPE at 550 mg/kg/day showed a lower level of total bilirubin compared to both control groups. 

#### 3.3.5. Histopathological Evaluation of PPE Treatment

For all rats, remarkable macroscopic lesions were not observed during the autopsy process. Histopathological evaluation also indicated that remarkable lesions were not found in most organs of the rats treated with PPE. Minor lesions were found on specific organs, as summarized in Tables 8 and 9. 

 For male rats, the groups treated with 11, 110, and 550 mg/kg/day of PPE showed significantly higher incidence of cortical fat infiltration in the adrenal glands when compared to the water control group. Only the group receiving the highest dose of PPE showed a significant increase in bile duct hyperplasia compared to both control groups. In the female rats, hyperplasia of the mammary gland was found in both control groups but not in the 11 and 110 mg/kg/day PPE-treated groups. The significant lesion in the kidney was congested tubules in female rats given 1,100 mg/kg/day. Furthermore, dilated tubules were also observed in some of the female rats receiving 550 and 1,100 mg/kg/day of PPE.

## 4. Discussion

The partially purified plaunotol extract (PPE) used in this acute and chronic toxicity study was prepared from the leaves of *C. stellatopilosus*. It was extracted by 95% ethanol under reflux, followed by liquid-liquid partition with hexane to remove nonpolar impurities. The major constituents of the resulting ethanol extract of PPE appeared to be 43% w/w of plaunotol with a similar amount of a plaunotol-like compound, presumably a fatty acid-plaunotol conjugate. Kitazawa et al. in 1982 reported on the presence of various geranylgeraniol derivatives in the extracts of *C. stellatopilosus*, including plaunotol (18-hydroxyl geranylgeraniol) and a number of fatty acid esters of geranylgeraniol [15]. 

The PPE obtained from processing was expected to be usable as a natural health product with its therapeutic benefits similar to that of plaunotol. This acyclic diterpenoid, originally found to possess antigastric ulcer activity, has continuously been reported to have bactericidal activity against *Helicobacter pylori* and potential as an anticancer agent [5, 16–18]. In this study, PPE was calculated to contain an equivalent amount of plaunotol for antigastric ulcer treatment in humans (4.8 mg/kg/day), which is equivalent to a dose of 11 mg/kg/day of PPE [11]. Our results obtained from the acute toxicity studies revealed that the tolerated dose of PPE was approximately 2.5 g/kg, or 200-fold higher than the recommended therapeutic dose. With this high dose, death was not observed in ICR mice, but mild diarrhea was the only observed side effect. In referring to toxicity classification, PPE can be categorized as a practical, non-toxic agent [19], with an LD_50_ of 10.25 g/kg. This LD_50_ value and the observed clinical signs following PPE treatment were not different from the data on pure plaunotol, which has previously been reported to have LD_50_ values of 8.8 and 8.1 g/kg for male and female mice, respectively [16]. 

Based on the LD_50_ of 10.25 g/kg, PPE was administered by oral gavage at doses of 11, 110, 550, and 1,100 mg/kg/day to a total of 180 Wistar rats, male and female, for chronic oral toxicity evaluation. Clinical and health status of the rats were observed for 6 months during treatment. The results showed that PPE did not suppress food consumption in all groups of rats, although significant body weight loss was observed in male rats treated with 1,100 mg/kg/day. These results suggest that the high concentration of acyclic diterpenoids in PPE may increase the metabolic rate in male rats, as reported previously [20]. However, this weight loss was only observed in male rats, and not in females. The differential responses may be gender dependent. In terms of organ weights, the relative weights of most organs were increased due to PPE treatment mainly in the male rats, whereas relative weights of the liver, kidney, and stomach were increased in both male and female rat groups. The latter may be due to responses at the subcellular level, such as stimulation of proteins or enzymes production [21]. Plaunotol exposure has been shown to induce COX2 and PGE_2_ expression in gastric epithelial cells of rats [6]. Furthermore, the observed congestion of blood in renal venules, as well as the dilated renal tubules, may have affected the relative weight of kidneys in both male and female rats.

The altered hematological values were not significantly different from reference values for normal rats [21, 22], except the increased platelet number in the highest dose-treated group. The underlying mechanism for this observed increase in platelet levels by plaunotol and the fatty acid-plaunotol conjugate in PPE remains to be understood. 

The significant increase in ALP levels caused by disorders of the hepatobiliary duct and osteoblast activity has been reported previously [23]. This response was observed in male rats treated with PPE at 1,100 mg/kg/day and in female rats treated with PPE at 550 and 1,100 mg/kg/day. Although the geranylgeraniol compound has been reported to have influence on osteoblast formation [24], abnormalities in bone formation were not observed in any of the rats. Additionally, the albumin values were increased male rats treated with 110 to 1,100 mg/kg/day and female rats given 550 and 1,100 mg/kg/day. This may be due to the stimulation of insulin, growth hormone, and corticosteroids [25]. The relationship between the plaunotol extract and hormonal expression is still unclear and requires further studies [26].

 The values of AST, uric acid, and bilirubin were not related to the administered concentrations of PPE, so these alterations may not be due to the plaunotol extract. In contrast, the increase in BUN values in female rats treated with 550 and 1,100 mg/kg/day may be the result of the high reabsorption of enlarged renal tubules. 

Recently, Gutierrez et al. in 2011 demonstrated an interesting effect of geranylgeraniol derivatives on blood glucose levels in diabetic rats. Specifically, hyperglycemia was apparent in rats treated with geranylgeranyl octadecanoate [20]. While the hydrophilic derivatives, such as geranylgeranyl acetate, lower blood glucose levels [20], our present study showed the hyperglycemic activity of PPE in male rats given doses of 11 to 550 mg/kg/day and in female rats treated with 110 to 1,100 mg/kg/day. The lowered blood glucose was only observed following treatment with 1,100 mg/kg/day in male rats. The varied effect on blood glucose may be gender dependent following treatment with the geranylgeraniol derivatives including plaunotol and its fatty acid conjugate in PPE. 

Although there were altered levels of chloride, potassium, protein, and creatinine in the blood, these biochemical values are still within the normal clinical ranges [22]. The increased cholesterol levels in female rats treated with 550 and 1,100 mg/kg/day may be caused by the high dose of PPE that was administered. The essential role geranylgeraniol derivatives play in cholesterol synthesis has been reported previously [27, 28].

It is known that, following oral administration, plaunotol is metabolized via the liver and excreted through bile and urine [11]. Thus, large doses of PPE may result in pathological changes in liver and kidney cells. Histopathological evaluation revealed apparent bile duct hyperplasia in both male and female rats treated with 1,100 mg/kg/day. Congestion and dilation of renal tubules were observed at doses of 1,100 mg/kg/day of PPE in female rats. Incidence of fat accumulation in the adrenal gland in male rats and mammary gland hyperplasia in female rats were not related to the concentration of PPE and therefore may not be due to treatment with the extract. 

## 5. Conclusion

This research demonstrated the safety of using PPE, supporting the traditional use of plaunotol in the form of crude extracts. It was apparent that PPE treatment at the therapeutic dose did not induce any lethal pathological or clinical signs following chronic exposure. Therefore, it may be possible to develop this extract as a human healthcare product. Nevertheless, administration of high doses of PPE over a long period of time may cause deterioration of the liver and renal tissue, including increased blood platelet levels. Therefore, hematological and biochemical values should be monitored during use of high doses of the extract over a long period of time.

## Figures and Tables

**Table 1:** Observed acute toxicity symptoms and mortality in mice receiving different single doses of PPE.

Dose (g/kg)	Symptoms/Time (Onset to recovery)				Mortality	
	Dyspnea	Dullness	Abdominal cramp	Diarrhea	Death/Total	Latency period
Control	None	None	None	None	0/10	—
2.5	None	None	None	6–72 h	0/10	—
5	None	None	None	6–72 h	0/10	—
10	15 min–24 h	15 min–24 h	15 min–24 h	6–96 h	4/10	24 h
20	15 min–24 h	15 min–24 h	15 min–24 h	6–72 h	10/10	24–72 h

**Table 2:** Relative organ weight (g/1000 g of body weight) and body weight (g) of male rats receiving PPE for 6 months.

Organs	Dose of PPE administered (mg/kg/day)					
	Control	Tragacanth	11	110	550	1100
	*n* = 15	*n* = 15	*n* = 15	*n* = 14^a^	*n* = 15	*n* = 15
Brain	3.88 ± 0.39	3.72 ± 0.33	3.78 ± 0.22	3.91 ± 0.31	4.21 ± 0.26^#^	4.55 ± 0.28^∗,#^
Heart	2.63 ± 0.28	2.74 ± 0.12	2.68 ± 0.28	2.89 ± 0.13^#^	3.40 ± 0.25^∗,#^	3.31 ± 0.27^∗,#^
Lung	3.07 ± 0.31	3.18 ± 0.19	3.45 ± 1.04	3.31 ± 0.19	3.70 ± 0.29^∗,#^	4.02 ± 0.43^∗,#^
Liver	23.86 ± 2.02	24.38 ± 1.82	25.57 ± 1.68	33.65 ± 3.62^∗,#^	49.65 ± 2.37^∗,#^	59.81 ± 3.17^∗,#^
Stomach	4.01 ± 0.40	3.81 ± 0.50	4.06 ± 0.39	4.53 ± 0.47^#^	5.20 ± 0.43^∗,#^	5.64 ± 0.39^∗,#^
Spleen	1.68 ± 0.20	1.55 ± 0.16	1.60 ± 0.15	1.77 ± 0.18^#^	2.14 ± 0.29^∗,#^	2.18 ± 0.14^∗,#^
Right kidney	2.33 ± 0.17	2.27 ± 0.21	2.38 ± 0.21	2.71 ± 0.18^∗,#^	3.12 ± 0.25^∗,#^	3.43 ± 0.31^∗,#^
Left kidney	2.21 ± 0.19	2.20 ± 0.22	2.22 ± 0.22	2.63 ± 0.20^∗,#^	3.04 ± 0.22^∗,#^	3.35 ± 0.24^∗,#^
Right testis	5.52 ± 0.53	5.16 ± 0.70	5.40 ± 0.43	5.48 ± 0.52	6.25 ± 0.55^∗,#^	6.61 ± 0.49^∗,#^
Left testis	5.60 ± 0.64	5.24 ± 0.82	5.41 ± 0.44	5.50 ± 0.56	6.27 ± 0.58^#^	6.62 ± 0.46^∗,#^
Right adrenal	0.071 ± 0.01	0.071 ± 0.02	0.067 ± 0.01	0.080 ± 0.01	0.088 ± 0.01^∗,#^	0.080 ± 0.01
Left adrenal	0.075 ± 0.01	0.082 ± 0.01	0.077 ± 0.02	0.087 ± 0.02	0.095 ± 0.01*	0.092 ± 0.02*
Bladder	0.328 ± 0.06	0.327 ± 0.06	0.313 ± 0.06	0.365 ± 0.09	0.418 ± 0.04^∗,#^	0.368 ± 0.12
Initial body weight	113.89 ± 4.57	113.25 ± 4.70	114.19 ± 5.91	114.46 ± 8.98	113.15 ± 4.70	113.50 ± 6.34
Final body weight	533.07 ± 55.27	573.47 ± 52.91	569.32 ± 42.54	545.16 ± 49.74	506.27 ± 30.97^#^	461.69 ± 29.35^∗,#^

The values are expressed as the mean ± SD.

*Significantly different from water (*P* < 0.05).

^#^Significantly different from 0.5% tragacanth (*P* < 0.05).

^a^Died from feeding extract into respiratory tract.

**Table 3:** Relative organ weight (g/1000 g body weight) and body weight (g) of female rats receiving PPE for 6 months.

Organs	Dose of PPE administered (mg/kg /day)					
	Control	Tragacanth	11	110	550	1100
	*n* = 15	*n* = 15	*n* = 15	*n* = 14^a^	*n* = 15	*n* = 15
Brain	6.88 ± 0.65	7.00 ± 0.62	6.82 ± 0.67	6.61 ± 0.54	6.72 ± 0.47	7.20 ± 0.39
Heart	3.30 ± 0.26	3.30 ± 0.33	3.27 ± 0.34	3.32 ± 0.20	3.55 ± 0.35	3.58 ± 0.23
Lung	4.58 ± 0.49	4.54 ± 0.50	4.49 ± 0.56	4.31 ± 0.31	4.62 ± 0.51	4.70 ± 0.30
Liver	27.08 ± 3.46	26.24 ± 2.93	25.60 ± 1.90	28.96 ± 3.38	44.80 ± 8.19^∗,#^	54.68 ± 2.79^∗,#^
Stomach	5.64 ± 0.80	5.58 ± 0.58	5.37 ± 0.65	5.61 ± 0.53	6.66 ± 1.00*	6.88 ± 0.60^∗,#^
Spleen	2.22 ± 0.24	2.28 ± 0.26	2.19 ± 0.23	2.22 ± 0.27	2.29 ± 0.16	2.26 ± 0.29
Right kidney	3.03 ± 0.20	3.00 ± 0.28	2.99 ± 0.33	3.01 ± 0.26	3.51 ± 0.60*	3.59 ± 0.28^∗,#^
Left kidney	2.86 ± 0.21	2.85 ± 0.21	2.85 ± 0.37	2.90 ± 0.19	3.43 ± 0.54^∗,#^	3.44 ± 0.29^∗,#^
Right adrenal	0.153 ± 0.02	0.145 ± 0.03	0.147 ± 0.03	0.130 ± 0.02	0.163 ± 0.06	0.136 ± 0.02
Left adrenal	0.155 ± 0.02	0.153 ± 0.03	0.156 ± 0.03	0.154 ± 0.03	0.170 ± 0.09	0.145 ± 0.02
Bladder	0.318 ± 0.04	0.321 ± 0.06	0.312 ± 0.06	0.339 ± 0.09	0.374 ± 0.08	0.389 ± 0.06
Uterus	2.31 ± 0.67	2.69 ± 1.05	2.53 ± 0.64	2.17 ± 0.64	2.55 ± 0.76	2.40 ± 0.58
Right ovary	0.279 ± 0.05	0.284 ± 0.07	0.277 ± 0.06	0.267 ± 0.07	0.309 ± 0.07	0.288 ± 0.06
Left ovary	0.291 ± 0.05	0.305 ± 0.09	0.303 ± 0.07	0.257 ± 0.07	0.303 ± 0.06	0.315 ± 0.09
Initial body weight	125.05 ± 14.22	122.79 ± 12.06	123.22 ± 11.99	124.06 ± 10.67	123.27 ± 14.54	121.77 ± 11.17
Final body weight	290.75 ± 29.24	283.54 ± 27.76	294.23 ± 30.84	301.79 ± 24.01	294.16 ± 22.49	275.07 ± 16.64

The values are expressed as the mean ± SD.

*Significantly different from water (*P* < 0.05).

^#^Significantly different from 0.5% Tragacanth (*P* < 0.05).

^a^Died from feeding extract into respiratory tract.

**Table 4:** Hematological values for male rats receiving PPE for 6 months.

Parameters	Dose of PPE administered (mg/kg/day)					
	Water	0.5% tragacanth	11	110	550	1100
	*n* = 15	*n* = 15	*n* = 15	*n* = 14^a^	*n* = 15	*n* = 15
Hematocrit (%)	31.23 ± 1.84	31.63 ± 1.82	31.47 ± 0.93	32.11 ± 1.80	32.82 ± 1.88	33.61 ± 1.83^∗,#^
RBC (×10^6^ cells/mm^3^)	9.66 ± 0.71	9.90 ± 0.51	9.70 ± 0.39	9.97 ± 0.67	10.33 ± 0.76	10.59 ± 0.75*
Hemoglobin (g/dL)	16.82 ± 0.96	16.90 ± 0.98	16.97 ± 0.53	17.12 ± 0.88	17.49 ± 0.98	17.82 ± 0.92*
MCV (*µ*m^3^/red cell)	53.85 ± 0.73	53.47 ± 0.55	53.94 ± 0.79	53.32 ± 0.89	53.31 ± 0.57	53.06 ± 0.70
MCH (pg/red cell)	17.44 ± 0.71	17.07 ± 0.49	17.51 ± 0.69	17.20 ± 0.56	16.96 ± 0.48	16.86 ± 0.55
MCHC (g/dL RBC)	32.39 ± 1.26	31.92 ± 0.99	32.46 ± 1.08	32.25 ± 1.10	31.83 ± 0.91	31.79 ± 1.05*
WBC (×10^3^ cells/mm^3^)	3.79 ± 1.29	3.97 ± 1.13	4.40 ± 1.65	4.87 ± 1.45	5.21 ± 1.62	4.45 ± 0.77
Neutrophil (%)	26.17 ± 5.81	29.36 ± 6.99	30.67 ± 13.68	27.59 ± 10.65	30.11 ± 11.32	25.31 ± 6.58
Eosinophil (%)	1.52 ± 0.66	1.33 ± 0.47	1.16 ± 0.42	1.00 ± 0.40	0.77 ± 0.25^∗,#^	0.74 ± 0.21^∗,#^
Lymphocyte (%)	67.95 ± 9.14	66.73 ± 6.23	65.06 ± 14.21	69.44 ± 9.91	67.66 ± 11.01	72.01 ± 6.47
Monocyte (%)	2.62 ± 3.39	1.62 ± 2.82	1.84 ± 2.76	0.98 ± 1.64	0.61 ± 0.29	0.58 ± 0.17
Basophil (%)	1.75 ± 1.95	0.95 ± 0.99	1.28 ± 1.25	0.98 ± 0.91	0.86 ± 0.39	1.35 ± 0.45
Platelet (×10^3^ cells/mm^3^)	1,020.57 ± 102.85	1,122.97 ± 118.94	1,052.83 ± 111.57	1,133.82 ± 140.14	1,297.57 ± 128.87^∗,#^	1,455.37 ± 205.83^∗,#^

The values are expressed as the mean ± SD.

*Significantly different from water (*P* < 0.05).

^#^Significantly different from 0.5% tragacanth (*P* < 0.05).

^a^Died from feeding extract into respiratory tract.

**Table 5:** Hematological values for female rats receiving PPE for 6 months.

Parameters	Dose of PPE administered (mg/kg/day)					
	Water	0.5% tragacanth	11	110	550	1100
	*n* = 15	*n* = 15	*n* = 15	*n* = 14^a^	*n* = 15	*n* = 15
Hematocrit (%)	30.86 ± 0.82	30.85 ± 1.40	30.88 ± 0.92	30.82 ± 1.28	31.86 ± 1.51	31.62 ± 1.44
RBC (×10^6^ cells/mm^3^)	8.93 ± 0.37	8.97 ± 0.52	8.98 ± 0.34	8.80 ± 0.38	9.29 ± 0.60	9.65 ± 0.37^∗,#^
Hemoglobin (g/dL)	16.60 ± 0.42	16.72 ± 0.60	16.79 ± 0.53	16.73 ± 0.64	17.00 ± 0.72	16.73 ± 0.74
MCV (*µ*m^3^/red cell)	53.77 ± 0.50	54.23 ± 0.72	54.40 ± 0.59	54.27 ± 0.52	53.37 ± 0.96	52.90 ± 0.55^∗,#^
MCH (pg/red cell)	18.61 ± 0.47	18.69 ± 0.58	18.71 ± 0.49	19.00 ± 0.47	18.34 ± 0.89	17.34 ± 0.53^∗,#^
MCHC (g/dL RBC)	34.60 ± 0.85	34.45 ± 0.83	34.38 ± 0.74	35.05 ± 0.74	34.33 ± 1.32	32.78 ± 0.93^∗,#^
WBC (×10^3^ cells/mm^3^)	1.95 ± 0.50	2.05 ± 0.61	2.40 ± 0.55	2.49 ± 0.78	2.56 ± 0.69	2.82 ± 1.04*
Neutrophil (%)	22.36 ± 4.49	25.03 ± 9.50	24.65 ± 8.51	19.86 ± 4.82	21.35 ± 11.41	20.94 ± 3.38
Eosinophil (%)	1.64 ± 0.56	1.50 ± 0.79	1.27 ± 0.70	1.20 ± 0.47	0.73 ± 0.34^∗,#^	0.74 ± 0.55*
Lymphocyte (%)	72.25 ± 5.08	71.95 ± 9.23	72.53 ± 8.55	76.97 ± 4.91	75.19 ± 12.68	75.80 ± 3.49
Monocyte (%)	1.94 ± 2.89	0.89 ± 0.30	0.93 ± 0.36	1.05 ± 0.65	1.43 ± 1.63	0.93 ± 0.67
Basophil (%)	0.83 ± 1.02	0.62 ± 0.41	0.63 ± 0.40	0.92 ± 0.46	1.29 ± 0.62^#^	1.59 ± 0.61^#^
Platelet (×10^3^ cells/mm^3^)	1,068.73 ± 121.33	1,039.23 ± 102.92	1,012.80 ± 71.82	1,015.57 ± 97.02	1,240.60 ± 106.72^∗,#^	1,334.80 ± 101.78^∗,#^

The values are expressed as the mean ± SD.

*Significantly different from water (*P* < 0.05).

^#^Significantly different from 0.5% tragacanth (*P* < 0.05).

^a^Died from feeding extract into respiratory tract.

**Table 6:** Biochemical values for male rats receiving PPE for 6 months.

Parameters	Dose of PPE administered (mg/kg/day)					
	Water	0.5% tragacanth	11	110	550	1100
	*n* = 15	*n* = 15	*n* = 15	*n* = 14^a^	*n* = 15	*n* = 15
ALP (U/L)	51.67 ± 9.03	48.13 ± 7.28	55.53 ± 22.60	55.29 ± 4.29^#^	70.53 ± 9.51^∗,#^	84.53 ± 14.39^∗,#^
ALT (U/L)	43.67 ± 15.99	38.47 ± 11.91	43.87 ± 23.19	33.71 ± 4.75	41.20 ± 7.63	43.93 ± 5.60
AST (U/L)	99.60 ± 33.66	86.33 ± 13.70	92.27 ± 39.18	74.29 ± 10.03	69.20 ± 6.94*	74.53 ± 15.57
Total protein (g/dL)	6.41 ± 0.18	6.67 ± 0.24	6.70 ± 0.18*	6.67 ± 0.22	6.84 ± 0.33*	6.66 ± 0.29
Albumin (g/dL)	4.51 ± 0.19	4.54 ± 0.09	4.61 ± 0.14	4.67 ± 0.23	5.03 ± 0.27^∗,#^	5.27 ± 0.24^∗,#^
Total bilirubin (mg/dL)	0.074 ± 0.03	0.070 ± 0.02	0.072 ± 0.02	0.104 ± 0.17	0.051 ± 0.01	0.071 ± 0.01
BUN (mg/dL)	18.75 ± 4.60	18.77 ± 3.11	16.71 ± 2.28	20.46 ± 3.13	22.59 ± 3.51^∗,#^	26.53 ± 4.98^∗,#^
Creatinine (mg/dL)	0.50 ± 0.08	0.52 ± 0.07	0.47 ± 0.06	0.47 ± 0.07	0.48 ± 0.07	0.49 ± 0.07
Glucose (mg/dL)	208.59 ± 36.28	265.05 ± 59.11	285.36 ± 36.34*	286.70 ± 63.09*	271.40 ± 51.29*	177.17 ± 34.34^∗,#^
Uric acid (mg/dL)	4.07 ± 1.18	5.43 ± 1.65	5.90 ± 1.33*	5.26 ± 1.33	6.23 ± 1.49*	4.73 ± 0.90
Triglyceride (mg/dL)	74.91 ± 21.16	81.59 ± 27.43	82.31 ± 25.37	99.32 ± 45.27	77.24 ± 26.90	37.45 ± 16.70^∗,#^
Cholesterol (mg/dL)	62.95 ± 10.31	75.38 ± 13.26	73.28 ± 14.85	84.36 ± 28.95	91.62 ± 19.62*	86.79 ± 16.99*
Sodium	143.20 ± 1.15	143.20 ± 1.26	143.27 ± 1.33	143.00 ± 0.88	143.40 ± 1.12	142.67 ± 1.05
Potassium	7.41 ± 1.02	7.40 ± 0.65	7.18 ± 0.66	6.93 ± 0.69	7.32 ± 0.56	7.67 ± 0.74
Chloride	105.00 ± 1.41	103.93 ± 1.49	103.20 ± 1.86	102.50 ± 0.85*	102.40 ± 1.68*	103.27 ± 1.67

The values are expressed as the mean ± SD.

*Significantly different from water (*P* < 0.05).

^#^Significantly different from 0.5% tragacanth (*P* < 0.05).

^a^Died from feeding extract into respiratory tract.

**Table 7:** Biochemical values for female rats receiving PPE for 6 months.

Parameters	Dose of PPE administered (mg/kg/day)					
	Water	0.5% tragacanth	11	110	550	1100
	*n* = 15	*n* = 15	*n* = 15	*n* = 14^a^	*n* = 15	*n* = 15
ALP (U/L)	22.93 ± 8.00	25.07 ± 7.29	23.80 ± 5.72	21.62 ± 7.68	22.38 ± 6.75	47.33 ± 12.97^∗,#^
ALT (U/L)	27.33 ± 4.45	28.60 ± 9.05	26.20 ± 4.99	23.54 ± 4.96	25.07 ± 9.76	29.00 ± 3.68
AST (U/L)	82.67 ± 9.77	87.40 ± 17.22	86.73 ± 11.74	75.15 ± 13.72	63.93 ± 6.57^∗,#^	73.60 ± 10.54
Total protein (g/dL)	6.63 ± 0.26	6.49 ± 0.28	6.51 ± 0.35	6.72 ± 0.33	6.99 ± 0.29^∗,#^	7.14 ± 0.27^∗,#^
Albumin (g/dL)	4.91 ± 0.20	4.82 ± 0.19	4.92 ± 0.26	5.15 ± 0.29^#^	5.49 ± 0.70^∗,#^	5.81 ± 0.14^∗,#^
Total bilirubin (mg/dL)	0.090 ± 0.02	0.098 ± 0.02	0.093 ± 0.02	0.089 ± 0.02	0.077 ± 0.01^#^	0.087 ± 0.02
BUN (mg/dL)	20.62 ± 5.44	21.91 ± 5.98	22.73 ± 4.99	25.23 ± 5.97	27.46 ± 5.99*	33.39 ± 7.34^∗,#^
Creatinine (mg/dL)	0.49 ± 0.08	0.50 ± 0.10	0.51 ± 0.09	0.53 ± 0.13	0.55 ± 0.09	0.68 ± 0.13^∗,#^
Glucose (mg/dL)	120.53 ± 16.41	110.66 ± 20.68	113.85 ± 12.41	138.09 ± 24.65^#^	156.02 ± 33.38^∗,#^	136.70 ± 21.60^#^
Uric acid (mg/dL)	3.15 ± 0.84	3.22 ± 0.55	2.83 ± 0.55	3.18 ± 0.65	4.16 ± 1.48	3.83 ± 1.16
Triglyceride (mg/dL)	42.47 ± 9.81	40.74 ± 9.16	40.71 ± 6.06	40.20 ± 10.36	41.81 ± 7.07	33.94 ± 6.50
Cholesterol (mg/dL)	65.30 ± 25.78	59.60 ± 15.74	54.69 ± 10.64	74.42 ± 20.54	89.14 ± 17.89^∗,#^	115.28 ± 26.26^∗,#^
Sodium	142.40 ± 1.18	141.53 ± 1.55	142.20 ± 1.01	142.31 ± 1.11	141.93 ± 0.88	142.20 ± 1.26
Potassium	7.38 ± 0.83	7.66 ± 1.06	7.13 ± 0.56	6.74 ± 0.88	6.81 ± 0.65	6.53 ± 0.68^#^
Chloride	106.60 ± 1.45	105.80 ± 1.47	106.07 ± 0.96	105.15 ± 1.41	103.27 ± 2.71^∗,#^	103.93 ± 1.16^∗,#^

The values are expressed as the mean ± SD.

*Significantly different from water (*P* < 0.05).

^#^Significantly different from 0.5% tragacanth (*P* < 0.05).

^a^Died from feeding extract into respiratory tract.

**Table 8:** Histopathological results of male rats receiving PPE for 6 months.

Organs	Microscopic findings	Dose of PPE administered (mg/kg/day)					
		Water	0.5% tragacanth	11	110	550	1100
Lung	BALT proliferation	5/15	5/15	2/15	5/14	4/15	5/15
Liver	Centrilobular fatty degeneration	0/15	2/15	2/15	1/14	2/15	0/15
	Centrilobular hydropic degeneration	0/15	0/15	2/15	1/14	0/15	0/15
	Bile ductule hyperplasia	0/15	0/15	0/15	0/14	1/15	10/15^∗,#^
Small intestine	GALT proliferation in submucosa	1/15	1/15	6/15	2/14	1/15	3/15
Large intestine	GALT proliferation in submucosa	1/15	1/15	0/15	1/14	2/15	3/15
Adrenal gland	Cortical fatty infiltration	0/15	2/15	6/15*	6/14*	4/15*	0/15

The results were expressed as the number of rats with pathological findings per total number of rats treated.

*Significantly different from water (*P* < 0.05).

^#^Significantly different from 0.5% tragacanth (*P* < 0.05).

BALT: bronchiole-associated lymphoid tissue; GALT: gut-associated lymphoid tissue.

**Table 9:** Histopathological results of female rats receiving PPE for 6 months.

Organs	Microscopic findings	Dose of PPE administered (mg/kg/day)					
		Water	0.5% tragacanth	11	110	550	1100
Lung	BALT proliferation	4/15	2/15	3/15	5/15	1/15	2/15
Liver	Bile ductule hyperplasia	0/15	0/15	0/15	0/15	1/15	2/15
	Dilated sinusoid and congestion	0/15	0/15	0/15	0/15	2/15	0/15
Kidney	Congestion	0/15	0/15	1/15	0/15	2/15	4/15^∗,#^
	Dilated tubules	0/15	0/15	0/15	0/15	1/15	3/15
Mammary gland	Glandular hyperplasia	4/15	4/15	0/15^∗,#^	0/15^∗,#^	1/15	6/15
Adrenal gland	Medullary congestion	2/15	3/15	3/15	3/15	1/15	2/15

The results were expressed as the number of rats with pathological findings per total number of rats treated, 1100: the satellite group.

*Significantly different from water (*P* < 0.05).

^#^Significantly different from 0.5% tragacanth (*P* < 0.05).

BALT: bronchiole-associated lymphoid tissue; GALT: gut-associated lymphoid tissue.
